# Transformer-based skeletal muscle deep-learning model for survival prediction in gastric cancer patients after curative resection

**DOI:** 10.1007/s10120-025-01614-w

**Published:** 2025-04-15

**Authors:** Qiuying Chen, Lian Jian, Hua Xiao, Bin Zhang, Xiaoping Yu, Bo Lai, Xuewei Wu, Jingjing You, Zhe Jin, Li Yu, Shuixing Zhang

**Affiliations:** 1 Department of Radiology, The First Affiliated Hospital, Jinan University, No. 613, Huangpu West Road, Tianhe District, Guangzhou, Guangdong 510627 People’s Republic of China; 2 Graduate College, Jinan University, Guangzhou, Guangdong People’s Republic of China; 3 Department of Radiology, Hunan Cancer Hospital, The Affiliated Cancer Hospital of Xiangya School of Medicine, Central South University, Changsha, Hunan People’s Republic of China; 4 Department of Hepatobiliary and Intestinal Surgery, Hunan Cancer Hospital and the Affiliated Cancer Hospital of Xiangya School of Medicine, Central South University, Changsha, Hunan People’s Republic of China; 5 Department of Radiology, Third Xiangya Hospital, Central South University, Changsha, Hunan People’s Republic of China

**Keywords:** Gastric cancer, Skeletal muscle, Sarcopenia, Survival, Deep-learning model

## Abstract

**Background:**

We developed and evaluated a skeletal muscle deep-learning (SMDL) model using skeletal muscle computed tomography (CT) imaging to predict the survival of patients with gastric cancer (GC).

**Methods:**

This multicenter retrospective study included patients who underwent curative resection of GC between April 2008 and December 2020. Preoperative CT images at the third lumbar vertebra were used to develop a Transformer-based SMDL model for predicting recurrence-free survival (RFS) and disease-specific survival (DSS). The predictive performance of the SMDL model was assessed using the area under the curve (AUC) and benchmarked against both alternative artificial intelligence models and conventional body composition parameters. The association between the model score and survival was assessed using Cox regression analysis. An integrated model combining SMDL signature with clinical variables was constructed, and its discrimination and fairness were evaluated.

**Results:**

A total of 1242, 311, and 94 patients were assigned to the training, internal, and external validation cohorts, respectively. The Transformer-based SMDL model yielded AUCs of 0.791–0.943 for predicting RFS and DSS across all three cohorts and significantly outperformed other models and body composition parameters. The model score was a strong independent prognostic factor for survival. Incorporating the SMDL signature into the clinical model resulted in better prognostic prediction performance. The false-negative and false-positive rates of the integrated model were similar across sex and age subgroups, indicating robust fairness.

**Conclusions:**

The Transformer-based SMDL model could accurately predict survival of GC and identify patients at high risk of recurrence or death, thereby assisting clinical decision-making.

**Supplementary Information:**

The online version contains supplementary material available at 10.1007/s10120-025-01614-w.

## Introduction

According to Global Cancer Statistics 2020, gastric cancer (GC) is the fifth most common cancer and the fourth leading cause of cancer-related death worldwide [1]. Similar to most solid tumors, disease progression in GC is usually associated with the degree of anorexia and underlying metabolic alterations. Recently, body composition analysis has been successful in nutritional evaluation [2–4], further supporting the argument for such measures to become part of clinical practice. Sarcopenia is a widely studied body composition parameter often defined as the loss of muscle mass and strength. It is closely associated with poor clinical outcomes, including increased disability, chemotherapy toxicity, postoperative complications, and shorter survival [5–9]. Sarcopenia is prevalent in patients with GC, ranging from 7 to 70% [10]. Accurate quantification of skeletal muscle is required for accurate diagnosis of sarcopenia [11]. Computed tomography (CT) is commonly used to measure skeletal muscle mass [12]. Specifically, the CT image of the third lumbar vertebra (L3) is significantly correlated with whole-body muscle [13, 14]. However, the diagnostic criteria for sarcopenia vary with race, sex, age, and disease status, and there is currently no unified global standard, thus hampering research and development in the field.

Using skeletal muscle size to assess the prognosis of GC has several limitations. It represents only one dimension of morphological change and does not include information on micro- and macroscopic changes in muscle architecture and composition. CT images not only display the quantity of skeletal muscle but also provide additional information that is difficult to discern through visual inspection. If multidimensional and deep-level features of skeletal muscle can be extracted from CT images, this could improve the value of skeletal muscle assessment in the prognostic evaluation of patients with GC.

Deep learning (DL) networks can complement single-modal images by providing additional information other than morphological changes. In particular, the Transformer-based DL model has achieved state-of-the-art performance across multiple tasks [15–17]. As a self-attention mechanism, the Transformer is not limited by the convolution operation. Therefore, it can better capture clear distant dependencies [18]. It also has other attractive features, such as easier scaling, greater robustness to high-frequency noise, and strong parallel computing ability [19]. Therefore, it has strong application potential in GC imaging analysis.

This study aimed to develop a Transformer-based skeletal muscle deep-learning (SMDL) model using preoperative L3-level skeletal muscle CT images to predict the survival of patients with GC after curative resection, compare the predictive performance of the model with other artificial intelligence (AI) models and traditional body composition parameters, and evaluate the model’s ability to identify patients at high risk of recurrence or death.

## Methods

### Study population and data sources

We retrospectively identified patients who underwent radical gastrectomy (R0 resection) between April 2008 and December 2020 from two centers. The inclusion criteria were: (1) age ≥ 18 years, (2) histologically confirmed GC, and (3) preoperative abdominal CT performed within 4 weeks. The exclusion criteria were: (1) preoperative anticancer treatment; (2) the presence of other malignancies; (3) history of gastrectomy; (4) chronic liver or kidney disease or other chronic wasting disease; (5) missing CT image, low-quality image, or CT scan range that did not reach the L3 level; and (6) skeletal muscle index (SMI) that could not be calculated, such as missing height data or edema affecting skeletal muscle measurement on CT images. A flowchart of the patient selection process is provided in Supplementary Fig. 1. A total of 1647 eligible patients were identified, including 1553 patients from Center 1 and 94 patients from Center 2. At Center 1, patients were randomly assigned to a training cohort (*n* = 1242) and an internal validation cohort (*n* = 311) in a 4:1 ratio. Patients from Center 2 served as the external validation cohort (*n* = 94) to assess the model’s generalizability.

All procedures followed were in accordance with the ethical standards of the Ethics Committee of the First Affiliated Hospital of Jinan University and national standards, and with the Helsinki Declaration of 1964 and later versions. The requirement for informed consent was waived due to the retrospective study design.

Data on patient characteristics were obtained from the medical records, including (1) general characteristics and clinical features: sex, age, body mass index (BMI), carcinoembryonic antigen (CEA) level, type of gastrectomy, and adjuvant chemotherapy; (2) tumor features: TNM stage, tumor differentiation grade, tumor location, and positive lymph node ratio; and (3) clinical outcomes: recurrence and death. Data were collected by experienced clinicians and reviewed by trained medical teams. Patients with more than 20% missing values were excluded, and the remaining missing values were coded as unknown variables for the analysis.

Each tumor was restaged according to the eighth edition of the American Joint Committee on Cancer staging system. The positive lymph node ratio was defined as the percentage of positive metastatic lymph nodes in the total number of dissected lymph nodes. The primary endpoint was recurrence-free survival (RFS), defined as the time from surgery to tumor recurrence or the last follow-up. The secondary endpoint was disease-specific survival (DSS), defined as the time from surgery to death due to GC or the last follow-up.

### Image acquisition and analysis

All patients underwent abdominal CT within four weeks of surgery. Detailed information on the CT protocols and variables is presented in Supplementary Table 1. The images were uploaded to a picture archiving and communication system.

We used DARWIN, an AI research platform for medical imaging (https://arxiv.org/abs/2009.00908), to segment the skeletal muscles semi-automatically. At the cross-section of L3, in which both transverse processes were visible, the areas of all skeletal muscles (the psoas, erector spinae, quadratus lumborum, transversus abdominis, external and internal obliques, and rectus abdominis) were measured, and the sum of these areas was calculated. The areas of all skeletal muscles were distinguishable from other tissue between − 29 and + 150 Hounsfield units on the CT scan [8]. This work was completed by two radiologists (with 5 years of experience in abdominal imaging) who were blinded to patient information. All delineations were reviewed and corrected by a senior radiologist (with 10 years of experience in abdominal imaging). The SMI was calculated by dividing the muscle area (cm^2^) by the patient’s height in meters squared (m^2^). The skeletal muscle density (SMD) was defined as the mean Hounsfield unit of all skeletal muscles. The BMI was calculated by dividing the patient’s weight (kg) by the square of the height in meters (m^2^).

Currently, no standardized cut-off point for low muscle mass exists for diagnosing sarcopenia. To evaluate the effect of sarcopenia on the prognosis of GC more comprehensively, we used different definitions of sarcopenia from several high-quality studies to classify patients (Supplementary Table 2) [7, 20, 21]. In addition, we defined sarcopenia using the optimal SMI cut-off point determined by the Youden index from the receiver operating characteristic (ROC) curve analysis.

### Construction of the SMDL model

DL models rely on big data samples. Insufficient data can lead to model overfitting or network training failure. Therefore, we used several methods to augment the training data, including mirroring, rotation, resizing, cropping, translation, and Gaussian noise, to improve the training results. Every enhancement strategy could stochastically transform data with internal parameters (e.g., rotation degree and noise level).

**Fig. 1:**
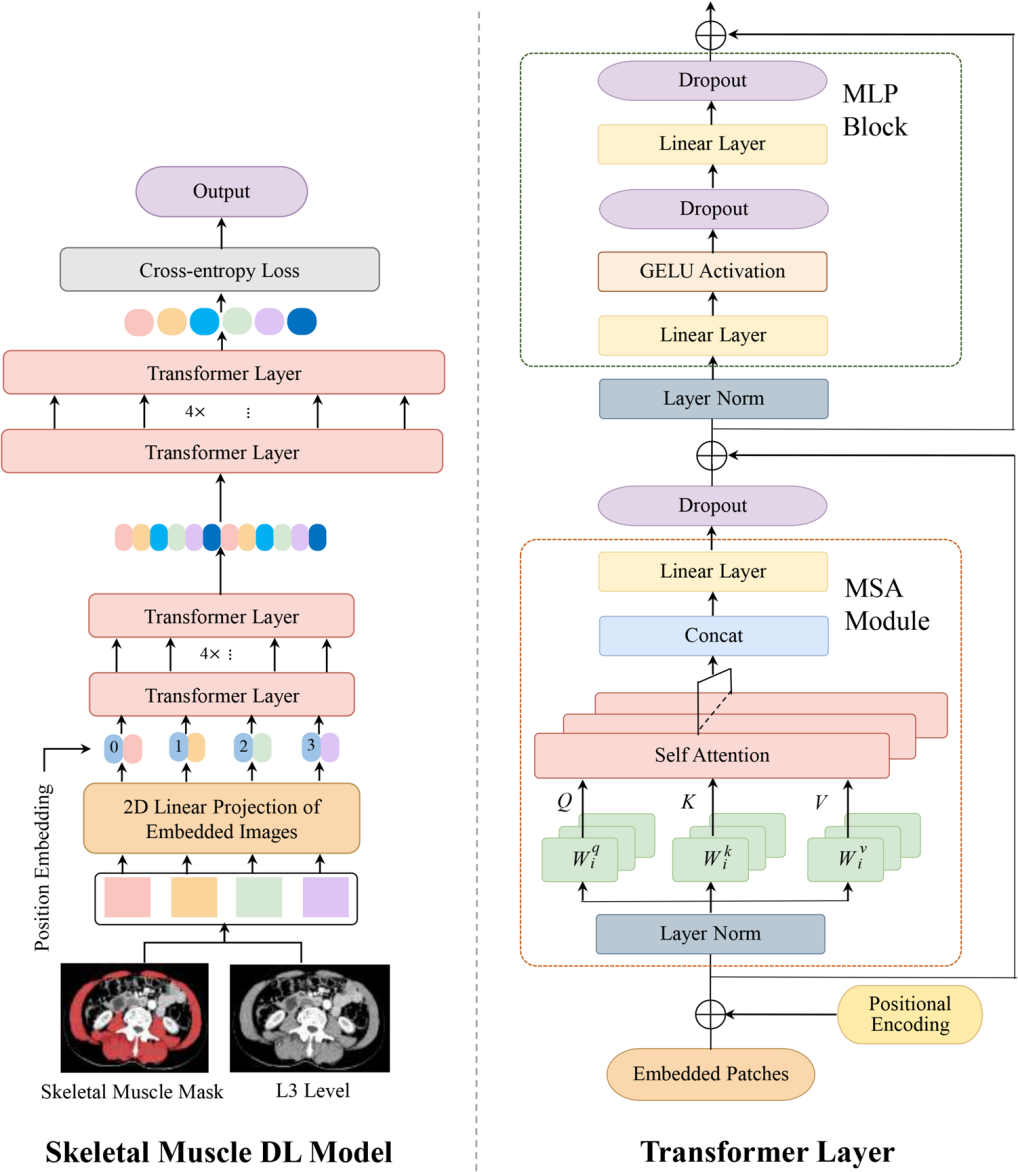
Schematic showing the features of the Transformer-based skeletal muscle deep-learning model

We used two-dimensional skeletal muscle images to build the DL model using Transformer as a network framework. Detailed information on the parameters of the SMDL model is presented in Fig. 1. The model was composed mainly of multiple Transformer layers. The Transformer effectively combined local and global information to improve the robustness of the features. Each Transformer layer was composed of a multi-head self-attention module and a multilayer perceptron block. The self-attention mechanism could not only aggregate the feature information of lesions on different scales but also correlate the context of input features effectively to aggregate the overall features of lesions and improve the expression of model features. During the training process, the model made predictions on the training data. The cross-entropy loss was calculated between these predictions and the ground truth labels. This loss value was then used by an optimization algorithm to update the model’s parameters in a way that minimizes the loss. By minimizing the cross-entropy loss, the model learned to make predictions that are closer to the ground truth, thus optimizing the model’s parameters. It resulted in the final output of the model, which was the risk score of recurrence and death in each patient with GC.

### Model assessment and comparisons

The performance of the SMDL model for predicting RFS and DSS was evaluated using the area under the ROC curve. To valid the superiority of the Transformer over other AI methods, we compared the Transformer-based SMDL model’s performance against common AI models, including the DenseNet, ResNet, and Rad-SVM models. The details of the three AI models are provided in Supplementary Methods. We also compared the predictive performance of the SMDL model with traditional body composition parameters (SMI, SMD, and BMI) to demonstrate the advantage of the DL method over traditional assessment methods in body composition analysis. The DeLong test was used to examine whether the differences were statistically significant. Unadjusted and multivariable Cox regression analyses were used to evaluate the effect of the model score on GC prognosis. Subgroup analysis of the association of the model score with survival was performed using stratified factors, including sex, age, CEA level, tumor location, adjuvant chemotherapy, TNM stage, and tumor differentiation grade.

To stratify patients into distinct risk groups, we applied the optimal cut-off point that maximized the Youden index in the training cohort. Specifically, patients with risk scores below the cut-off point were classified as low-risk, while those with scores equal to or above the cut-off point were designated as high-risk. The differences in the RFS and DSS between the two groups were compared using Kaplan–Meier survival analyses. Moreover, we compared the ability of prognostic stratification of the SMDL model, TNM stage, and different definitions of sarcopenia.

### Integration with clinicopathological variables

To demonstrate the incremental value of the SMDL signature to the clinicopathological risk factors for survival prediction, both clinical and integrated models were developed. The clinical model incorporated only the independent clinicopathological risk factors based on the multivariable Cox analysis. The integrated model incorporated the SMDL signature and the independent clinicopathological risk factors. To quantify the improvement of usefulness added by the SMDL signature, the net reclassification improvement (NRI) and integrated discrimination improvement (IDI) were calculated [22, 23]. The accuracy, sensitivity, specificity, positive predictive value (PPV), negative predictive value (NPV), and F1 score were measured to fully evaluate the predictive performance of the integrated model.

Specifically, we evaluated the fairness of the integrated model. Fairness addresses whether an algorithm treats subgroups equitably. Failure to account for age and sex differences between individuals leads to suboptimal results and discriminatory outcomes [24]. The composition of skeletal muscle may differ by age and sex, as skeletal muscle is generally larger in males than in females, and skeletal muscle is generally firmer in younger adults than in older adults. To quantify the fairness of the model in the context of clinical decision-making, we compared the false-negative and false-positive rates across different subgroups. The false-negative rate is the proportion of actual positive instances that are incorrectly classified as negative by the model. The false-positive rate is the proportion of actual negative instances that are incorrectly classified as positive. These two metrics could help us to identify subgroups that were under- or over-diagnosed using the DL model.

### Statistical analysis

SPSS (version 26.0), Python (version 3.8; https://www.python.org), and MedCalc (version 20.21; https://www.medcalc.org) were used for statistical analyses. Continuous variables are expressed as mean ± standard deviation and were compared using the one-way analysis of variance or the Kruskal–Wallis test. Categorical variables are presented as frequencies and percentages and were compared using the Chi-square test. To determine the independent prognostic factors for RFS and DSS, the variables that were significant in the unadjusted Cox regression analysis (*P* < 0.05) were included in the multivariable Cox regression analysis. Kaplan–Meier survival analysis was used to calculate RFS and DSS, and the log-rank test was used to evaluate the statistical significance of survival differences between groups. Two-tailed *P* < 0.05 were considered statistically significant.

## Results

### Patient characteristics

A total of 1647 patients (mean [standard deviation] age, 57.1 [10.8] years; 1075 [65.3%] male) were eligible for this study. The baseline characteristics for the training (*n* = 1242), internal (*n* = 311), and external validation (*n* = 94) cohorts are listed in Supplementary Table 3. During follow-up, 626 (38.0%) patients developed local or distant recurrence, with 479 cases (76.5%) occurring within the first 2 years. A total of 565 (34.2%) patients died of GC.

### Predictive performance of the SMDL model

**Fig. 2:**
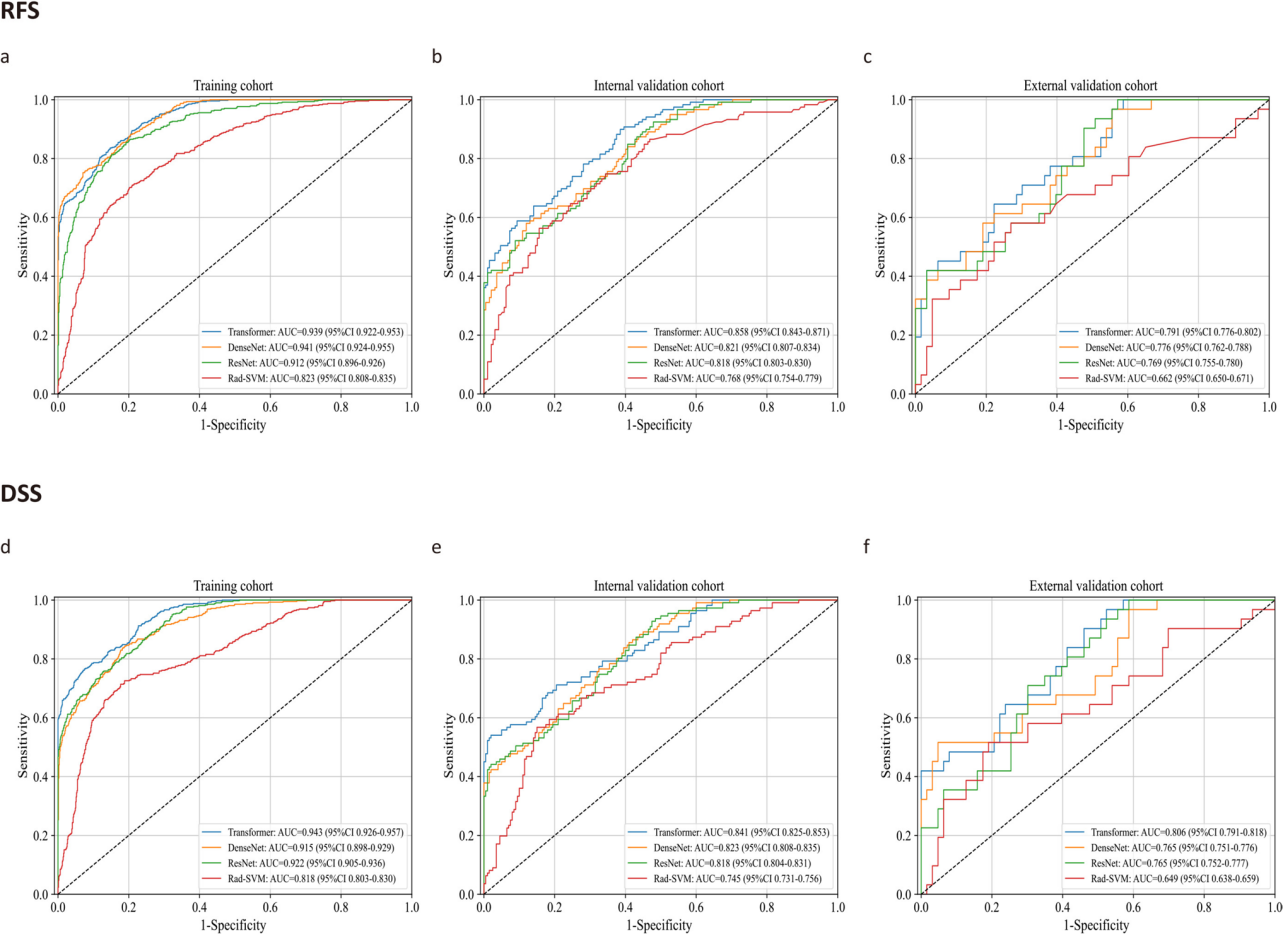
The predictive performance of several artificial intelligence models in the training, internal, and external validation cohorts

The predictive performance of different AI models in the three cohorts is presented in Fig. 2. Among the four AI models, the Transformed-based SMDL model exhibited the best discrimination ability. In the internal validation cohort, this model achieved an area under the curve (AUC) of 0.858 (95% confidence interval [CI] 0.843–0.871) for predicting RFS and an AUC of 0.841 (95% CI 0.825–0.853) for predicting DSS. Similarly, in the external validation cohort, the model exhibited an AUC of 0.791 (95% CI 0.776–0.802) for predicting RFS and an AUC of 0.806 (95% CI 0.791–0.818) for predicting DSS. These results highlight the superior predictive accuracy of the Transformer as a network framework.

**Fig. 3:**
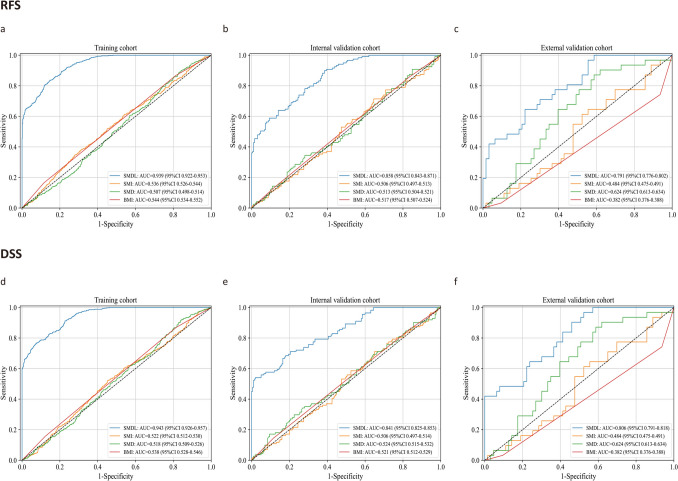
Comparisons of the predictive performance of the skeletal muscle deep-learning model and traditional body composition parameters in the training, internal, and external validation cohorts

In addition, the AUC values of the SMDL model for predicting RFS and DSS were significantly higher than that of the traditional body composition parameters (the AUC ranges from 0.382 to 0.624) across all three cohorts (DeLong test, all *P* < 0.001) (Fig. 3). The findings demonstrate that using a DL approach to interpret skeletal muscle images can provide more valuable information than traditional methods of predicting survival in patients with GC.

### Association between the model score and the prognosis of GC

Unadjusted and multivariable Cox regression analyses were performed to evaluate the association between the model score and the prognosis of GC patients. In the unadjusted analysis, higher model scores were significantly associated with worse RFS and DSS (Supplementary Tables 4–6). After adjusting for clinicopathological variables, the model score remained a robust independent predictor for both RFS (internal validation cohort: hazard ratio [HR] = 173.2, 95% CI 45.7–656.7, *P* < 0.001; external validation cohort: HR = 46.7, 95% CI 5.6–392.5, *P* < 0.001) and DSS (internal validation cohort: HR = 794.7, 95% CI 163.9–3854.3, *P* < 0.001; external validation cohort: HR = 329.8, 95% CI 27.8–3910.0, *P* < 0.001).

In the subgroup analysis, the effect of the model score on RFS (Supplementary Fig. 2) and DSS (Supplementary Fig. 3) was consistent across all subgroups. Notably, a stronger association between the model score and DSS was observed in patients with gastric antrum cancer, male sex, age ≥ 65 years, and stage II tumors (*P*-interaction = 0.003, 0.022, 0.007, and 0.021, respectively).

### Prognostic stratification using the SMDL model

**Fig. 4:**
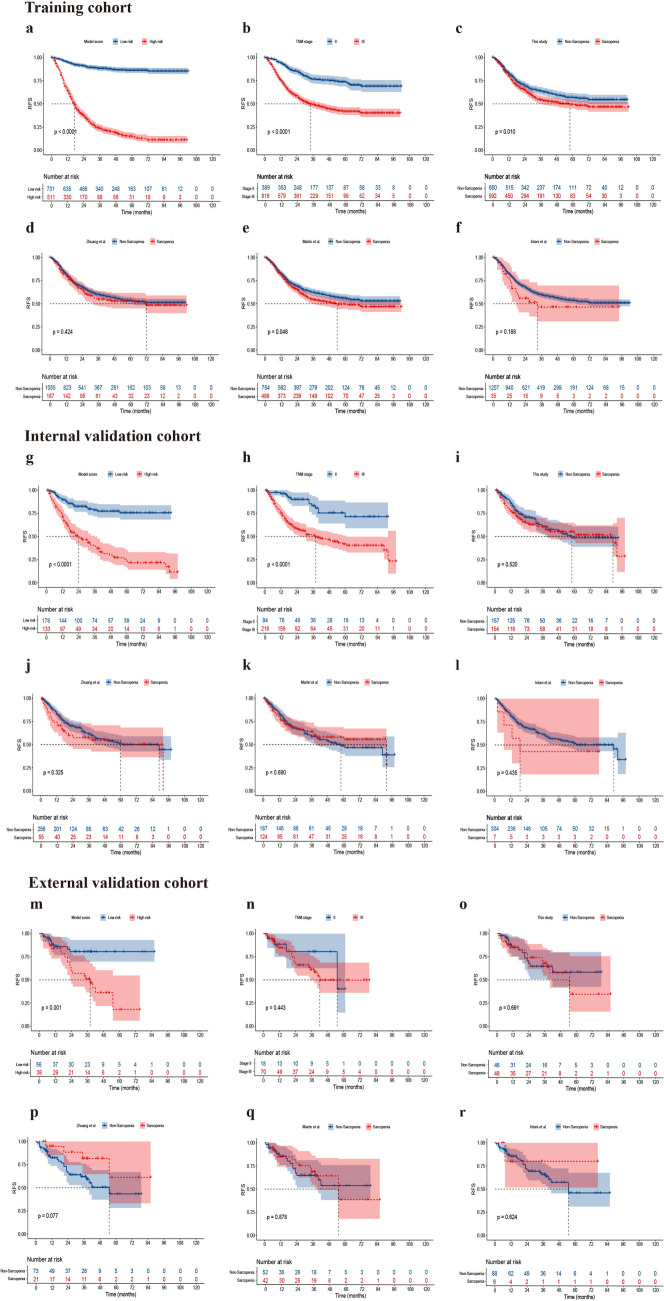
Kaplan–Meier analysis showing the difference in recurrence-free survival between different subgroups in the training, internal, and external validation cohorts

The patients were categorized into low- and high-risk groups according to the optimal cut-off point. In the internal validation cohort, the 5-year RFS was 23.4% and 75.5% in the high- and low-risk groups, respectively (HR: 4.3, 95% CI 2.9–6.2, *P* < 0.001; Fig. 4g), demonstrating that the SMDL model was able to accurately stratify patients with GC according to their prognosis, and was a better predictor of RFS than TNM stage (Fig. 4h). The model was also able to accurately distinguish patients with different prognosis in the external validation cohort (5-year RFS in high- vs. low-risk groups: 18.1% vs. 80.5%, HR: 3.4, 95% CI 1.7−7.0, *P* = 0.001; Fig. 4m). Moreover, although patients with sarcopenia, as defined by this study and the study by Martin et al. [7], had lower RFS than those without sarcopenia in the training cohort (*P* = 0.010 and *P* = 0.048; Fig. 4c and e), sarcopenia was not a significant predictor of survival in the internal and external validation cohorts (all *P* > 0.05; Fig. 4i, k, o, and q).

**Fig. 5:**
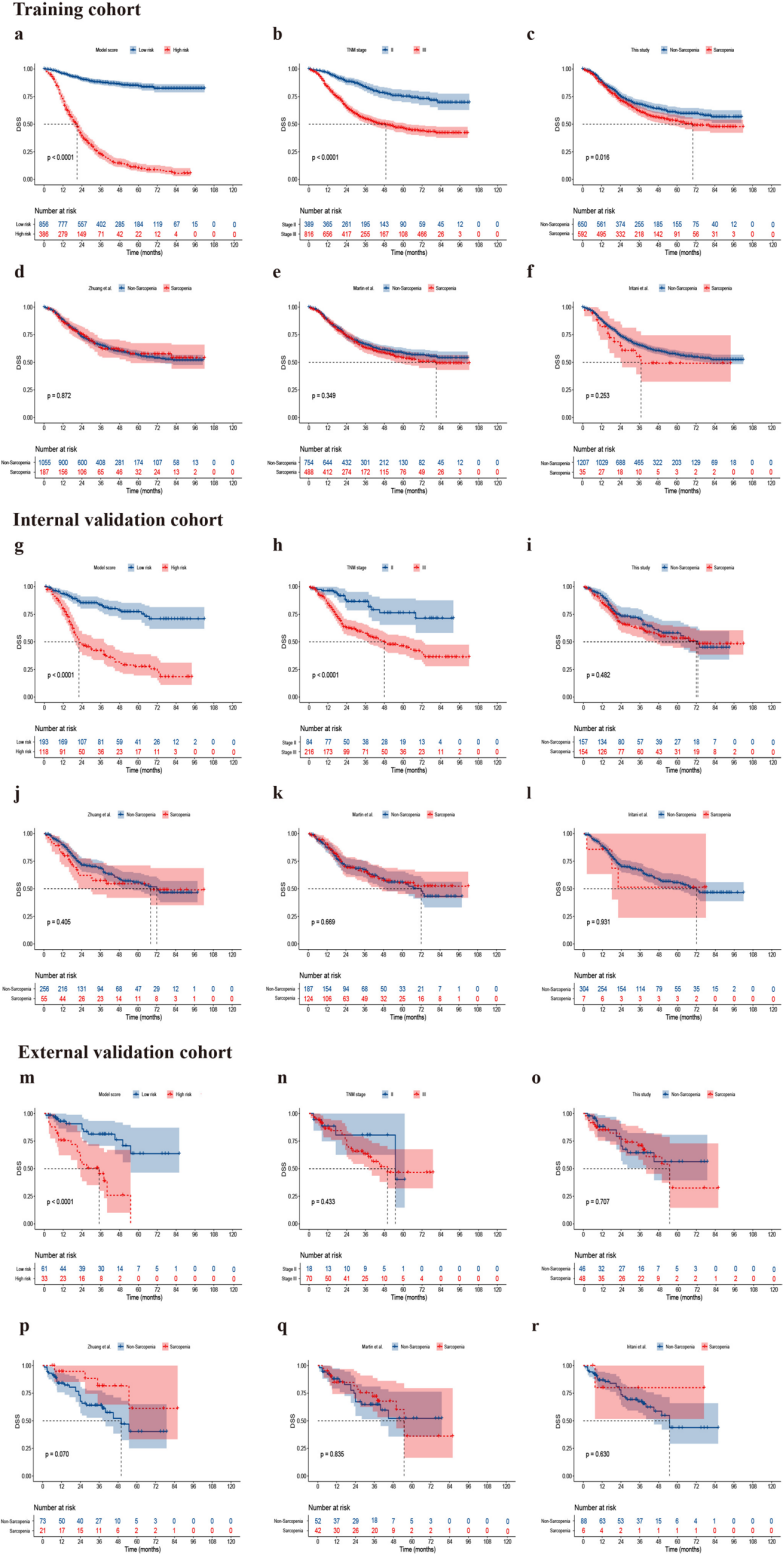
Kaplan–Meier analysis showing the difference in disease-specific survival between different subgroups in the training, internal, and external validation cohorts

The SMDL model was also effective in identifying patients at high risk of disease-specific death. The 5-year DSS was significantly lower in the high-risk group than in the low-risk group (internal validation cohort: 27.5% vs. 77.3%, HR: 4.5, 95% CI 3.0–6.7, *P* < 0.001; Fig. 5g; external validation cohort: 25.9% vs. 63.6%, HR: 3.9, 95% CI 1.8–8.6, *P* < 0.001; Fig. 5m). The sarcopenia defined by this study was associated with a lower DSS in the training cohort (HR: 1.3, 95% CI 1.0–1.5, *P* = 0.016; Fig. 5c), but not in the internal and external validation cohorts (*P* = 0.482 and *P* = 0.707; Fig. 5i and o).

### Integration with clinicopathological variables

In multivariable analysis, model score, CEA level, tumor location, N stage, T stage, positive lymph node ratio, and adjuvant chemotherapy were significant predictors of RFS (Supplementary Table 4). We then combined these variables and built an integrated model, which yielded higher accuracy than the clinical model, with AUCs of 0.876 (95% CI 0.860–0.887) and 0.823 (95% CI 0.809–0.836) for predicting RFS as well as AUCs of 0.859 (95% CI 0.837–0.875) and 0.822 (95% CI 0.807–0.839) for predicting DSS in the internal and external validation cohorts (Supplementary Tables 7 and 8). Furthermore, the inclusion of the SMDL signature in the clinical model yielded a total NRI of 0.273 (95% CI 0.156–0.382, *P* < 0.001) and 0.213 (95% CI 0.101–0.386, *P* < 0.001) for RFS and DSS, respectively, in the internal validation cohorts. Meanwhile, the IDI was 0.164 (95% CI 0.087–0.296) and 0.108 (95% CI 0.061–0.198) for RFS and DSS, respectively, in the internal validation cohorts. Similar results were observed in the external validation cohort, showing improved classification accuracy for survival outcomes (Supplementary Tables 7 and 8). The accuracy, sensitivity, specificity, PPV, NPV, and F1 score of the integrated model in each cohort are listed in Table 1.

**Table 1:** Predictive performance of the integrated model in the training, internal, and external validation cohorts

	RFS			DSS		
	Training cohort	Internal validation cohort	External validation cohort	Training cohort	Internal validation cohort	External validation cohort
AUC	0.984 (0.963–0.993)	0.876 (0.860–0.887)	0.823 (0.809–0.836)	0.966 (0.952–0.976)	0.859 (0.837–0.875)	0.822 (0.807–0.839)
Accuracy	0.930 (0.914–0.947)	0.781 (0.768–0.796)	0.723 (0.707–0.738)	0.888 (0.870–0.905)	0.755 (0.740–0.767)	0.712 (0.695–0.731)
Sensitivity	0.909 (0.887–0.926)	0.747 (0.733–0.765)	0.645 (0.631–0.661)	0.877 (0.860–0.896)	0.702 (0.691–0.715)	0.741 (0.728–0.756)
Specificity	0.942 (0.923–0.958)	0.802 (0.790–0.818)	0.761 (0.747–0.778)	0.894 (0.880–0.911)	0.785 (0.762–0.803)	0.698 (0.678–0.712)
PPV	0.907 (0.889–0.925)	0.700 (0.685–0.717)	0.571 (0.557–0.588)	0.811 (0.794–0.827)	0.644 (0.629–0.657)	0.547 (0.534–0.570)
NPV	0.944 (0.928–0.960)	0.836 (0.820–0.853)	0.813 (0.801–0.832)	0.933 (0.918–0.952)	0.826 (0.812–0.846)	0.846 (0.833–0.865)
*F*1 score	0.908 (0.891–0.933)	0.723 (0.708–0.739)	0.606 (0.592–0.618)	0.843 (0.829–0.857)	0.672 (0.651–0.693)	0.630 (0.619–0.648)

Data in parentheses are 95% confidence intervals (CIs)

*AUC* area under the curve, *DSS* disease-specific survival, *NPV* negative predictive value, *PPV* positive predictive value, *RFS* recurrence-free survival

### Fairness of the integrated model

We further calculated the false-negative and false-positive rates of the integrated model in different sex and age subgroups to evaluate the fairness. As shown in Table 2, the differences in the false-negative and false-positive rates between the different age and sex subgroups ranged from 0.2 to 8.1%, reflecting the acceptable fairness of the model.

**Table 2:** Comparison of the false-negative and false-positive rates of the integrated model in different age and sex subgroups

Subgroup	RFS		DSS	
	False-negative rate (%)	False-positive rate (%)	False-negative rate (%)	False-positive rate (%)
*Sex*				
Male	9.1	11.5	19.3	11.3
Female	10.5	11.7	16.8	12.0
*Age (years)*				
< 65	10.5	9.3	19.0	10.5
≥ 65	7.0	17.4	17.1	14.5

*DSS* disease-specific survival, *RFS* recurrence-free survival

## Discussion

A comprehensive sarcopenia assessment needs to consider not only muscle quantity and quality but also muscle strength and physical performance. According to the 2018 European Working Group on Sarcopenia in Older People 2 definition, sarcopenia is probable when low muscle strength is detected and is confirmed by the presence of low muscle quantity or quality [11]. Sarcopenia is considered severe when low muscle strength, low muscle quantity or quality, and low physical performance are all present. Screening for sarcopenia requires professional evaluation and involves time and energy to analyze grip strength and gait speed, which consumes human and medical resources. CT is an important tool for the analysis of muscle quantity and quality; however, the abundant muscle information on CT images is currently not fully used. In the present study, we extracted information from the preoperative L3-level skeletal muscle CT image using the Transformer-based DL model and explored the potential association between the skeletal muscle and the survival outcomes in patients with GC. Compared with a morphology-based model of sarcopenia, DL can capture the skeletal muscle heterogeneity in a non-invasive manner and use an automated feature-extraction algorithm to convert image data into high-resolution, minable image features. This can reduce the diagnostic bias caused by manual delineation of the skeletal muscle region and the subjective assessment of physical performance.

The rapid development of precision medicine imaging technology and ongoing advancements in image recognition technology and data algorithms have enabled the mining and analysis of medical image big data, which greatly expands the information volume of medical images. Researchers have reached beyond assessing the effect of skeletal muscle morphological changes on tumor prognosis and have extracted radiomic or DL features from skeletal muscle images for analysis and modeling, further improving the value of skeletal muscle in predicting the prognosis of patients with cancer. Chen et al. [25] established a radiomics-based diagnostic tool for sarcopenia. Compared with traditional definitions of sarcopenia, radiomic sarcopenia could effectively improve the prognostic accuracy of predicting survival and complications and shorten the time and steps of traditional screening, while reducing the subjectivity of sarcopenia assessment. Miao et al. [26] proposed a DL-radiomics approach to investigate the effects of muscle and fat on the survival of patients with breast cancer (BC). They found that appending the network features of muscle and fat at the T4 and T11 levels to the model significantly enhanced the accuracy of predicting distant metastasis of BC, providing a valuable biomarker for the early treatment of patients with BC. These studies demonstrated the effectiveness of AI technology in skeletal muscle image analysis and were used as a technical source of reference in the present study.

Some studies [21, 27–33] have explored the association between sarcopenia and postoperative outcomes in patients with GC, but the conclusions are inconsistent. Kamarajah et al. [10] conducted a systematic review and meta-analysis of these studies and found that the combined results showed that sarcopenia is associated with reduced overall survival, RFS, and DSS in patients undergoing gastrectomy. However, the assessment of postoperative outcomes was limited by differences in the methods used to assess body composition between studies, so the conclusions should be interpreted with caution. Therefore, the value of sarcopenia in the prognostic assessment of GC needs to be further explored. In the present study, we also analyzed the prognostic value of sarcopenia in patients with GC; however, its predictive ability was poor in all cohorts, with AUC values below 0.6. In addition, the Kaplan–Meier survival analyses showed that sarcopenia defined according to different criteria was not significantly associated with poorer survival outcomes. These results suggest that sarcopenia may not be an ideal prognostic indicator in patients with GC. In contrast, the prognostic value of the SMDL model was significantly higher than that of sarcopenia. The SMDL model could accurately distinguish patients with different prognoses and performed better than the TNM staging system. These findings demonstrate that applying a DL approach to analyze skeletal muscle images can provide more valuable information than traditional methods of predicting survival in patients with GC.

This study had some limitations. First, the study population is limited to Asians. The pathological subtypes, clinical features, and skeletal muscle status of patients with GC in Western countries may differ from those in the study cohort. Therefore, the generalizability of these findings to people living in different countries and regions should be rigorously tested. Second, we only analyzed the representative L3-level skeletal muscle CT images. In the future, we plan to analyze the whole abdominal skeletal muscle CT images to provide more heterogeneous information. Third, DL is a black box lacking interpretability. More acceptable and intuitive interpretation methods should be further investigated to facilitate the clinical application of DL models.

In conclusion, in this multicenter retrospective study, we developed and validated a Transformer-based SMDL model to accurately predict the survival outcomes of patients with GC after radical gastrectomy, using the preoperative L3-level skeletal muscle CT images. The predictive ability of the SMDL model was significantly better than that of other AI models and traditional body composition parameters. Compared with different definitions of sarcopenia and the TNM staging system, the SMDL model was more effective at identifying patients at high risk of recurrence or death, which could potentially help clinicians make appropriate choices of treatment regimens. However, this model warrants validation through prospective cohort studies.

## Supplementary Information

Below is the link to the electronic supplementary material.

Supplementary file1 (DOCX 606 kb)
