# Supplementary material for: Transformer-based skeletal muscle deep-learning model for survival prediction in gastric cancer patients after curative resection
Source: Gastric Cancer. 2025 Apr 15;28(4):684–95. doi: 10.1007/s10120-025-01614-w (PMC12174216; doi:10.1007/s10120-025-01614-w)
Supplement: Supplementary file 1 — Supplementary file1 (DOCX 606 kb) [file 10120_2025_1614_MOESM1_ESM.docx]

**Supplementary Materials**

- **Supplementary Methods**
- **Supplementary Tables**
- **Supplementary Figures**

**Supplementary Methods**

**ResNet model**

Firstly, the element-wise multiplication was performed between the binary mask and the original image to accurately extract the region of interest (ROI) of skeletal muscle, thereby eliminating the background noise. Subsequently, the extracted ROI was subjected to rigorous normalization, with all images resized to a uniform dimension of 224×224 pixels. To enhance the model's generalization ability, we applied a suite of data augmentation techniques, including random flipping, rotation, and brightness jittering, which effectively expanded the diversity of the training dataset and mitigated the risk of overfitting. The ResNet-101 was selected for its deep network structure, which is particularly adept at capturing high-level semantic features while simultaneously addressing the vanishing gradient problem inherent in very deep networks. The network was initialized with the pre-trained weights derived from the ImageNet, providing a strong baseline for feature extraction. Through the transfer learning, the network was fine-tuned to adapt to the classification tasks of gastric cancer survival. During the feature extraction, the input ROI images were processed through the deep residual blocks of the ResNet-101, where high-dimensional feature representations were extracted. These features were subsequently compressed into a 2048-dimensional feature vector via a Global Average Pooling (GAP) layer, which effectively aggregated spatial information while retaining critical semantic content. The feature vector was then passed through a fully connected (FC) layer followed by a Softmax activation function to produce the final prediction. In the training process, the cross-entropy loss function was used for optimization, and the Adam or SGD optimizer was used for gradient update to improve the training stability. The robustness of the model was evaluated using a 5-fold cross-validation, and the area under the curve (AUC) value was calculated.

**DenseNet model**

The procedure for ROI extraction and data preprocessing was identical to that described previously. Compared to the ResNet, the DenseNet introduces a more radical dense connectivity mechanism, where all layers are interconnected. Specifically, each layer receives the feature maps of all preceding layers as additional inputs. As a classification network, the DenseNet-121 leverages dense blocks for feature propagation and reuse, ensuring that the deep features are not lost due to vanishing gradients. The network parameters were initialized with the pre-trained weights from the ImageNet and fine-tuned through the transfer learning to adapt to the classification tasks of gastric cancer survival. The input images were processed through multiple convolutional layers and dense connectivity structures to extract the high-dimensional features, which were then compressed into a 1024-dimensional feature vector via a GAP layer. Finally, the feature vector was passed through a FC layer followed by a Softmax activation function to produce the prediction. In the training process, the cross-entropy loss function was used for optimization, and the Adam or SGD optimizer was used for gradient update to improve the training stability. The robustness of the model was evaluated using a 5-fold cross-validation, and the AUC value was calculated.

**Rad-SVM model**

Firstly, the element-wise multiplication was performed between the binary mask and the original image to accurately extract the ROI of skeletal muscle, thereby eliminating the background noise. Subsequently, the PyRadiomics software was used to extract and quantify the radiomics features from the ROI. A total of 1051 radiomics features were extracted, which mainly included three types of features: morphological features, histogram features, and texture features. The Principal Component Analysis (PCA) was employed for feature dimension reduction, thereby removing the redundant information while retaining the critical information. The dimension-reduced features were then fed into a Support Vector Machine (SVM) classifier for predicting recurrence-free survival and disease-specific survival. Various kernel functions, including Linear kernel, Radial Basis Function (RBF) kernel, and Polynomial kernel, were tested to explore the optimal classification performance. The experiment demonstrated that the RBF kernel could effectively exploit the non-linear relationships among the radiomic features, thereby improving the classification performance. Consequently, the RBF kernel-based SVM was selected as the classification model, with the hyperparameters (regularization parameter C and kernel parameter γ) optimized using the grid search to ensure the optimal performance. Additionally, a 5-fold cross-validation was employed during training to evaluate the robustness of the model, and the AUC value was calculated.

**Supplementary Tables**

**Supplementary Table 1** Information and parameters of CT scanners

|  |  | **Center 1** | **Center 2** |
| --- | --- | --- | --- |
| **CT system information** | CT system | 64-slice (Siemens Somatom Definition, Siemens); 256-slice (GE Revolution, GE Healthcare) | 64-slice (Toshiba Medical Systems, Japan); 64-slice (Somatom Force, Siemens Healthcare) |
| **CT scan parameters** | Tube voltage (kV) | 120 | 120 |
|  | Tube current (mA) | 30-350, 150-550 | Automatic exposure control |
|  | Rotation speed (s/r) | 0.5 | 0.5 |
|  | Image matrix (mm) | 512×512 | 512×512 |
|  | Field of view (mm) | 400×400 | 350×350 |
|  | Pitch | 0.6, 0.992 | 0.656, 0.7 |
|  | Width of detector (mm) | 38.4, 160 | 64, 192 |
|  | Slice thickness (mm) | 5 | 3 |
|  | Slice gap (mm) | 5 | 3 |

**Supplementary Table 2** Definitions of sarcopenia according to different literature

| **Literature** | **Definition of sarcopenia** |
| --- | --- |
| Martin et al. ^[7]^ | Male (BMI < 25.0 kg/m^2^) ≤ 43 cm^2^/ m^2^, Male (BMI > 25.0 kg/m^2^) ≤ 53 cm^2^/m^2^, Female ≤ 41 cm^2^/m^2^ |
| Iritani et al. ^[20]^ | Male ≤ 36.0 cm^2^/m^2^, Female ≤ 29.0 cm^2^/m^2^ |
| Zhuang et al. ^[21]^ | Male ≤ 40.8 cm^2^/m^2^, Female ≤ 34.9 cm^2^/m^2^ |
| The present study | Male ≤ 44.79 cm^2^/m^2^, Female ≤ 44.847 cm^2^/m^2^ |

**Supplementary Table 3** Clinicopathological features of patients with gastric cancer in the training, internal, and external validation cohorts

| **Clinicopathological feature** | **Training cohort**  **(n = 1242)** | **Internal validation cohort**  **(n = 311)** | **External validation cohort**  **(n = 94)** | ***P*-value** |
| --- | --- | --- | --- | --- |
| **Age**, years | 57.2 ± 11.0 | 56.4 ± 10.6 | 59.4 ± 10.2 | 0.036 |
| **Sex** |  |  |  | 0.913 |
| Male | 814 (65.5) | 201 (64.6) | 60 (63.8) |  |
| Female | 428 (34.5) | 110 (35.4) | 34 (36.2) |  |
| **BMI**, kg/m^2^ | 22.0 ± 3.1 | 21.9 ± 3.2 | 21.5 ± 2.6 | 0.276 |
| **CEA level** |  |  |  | <0.001 |
| Normal | 965 (77.7) | 249 (80.1) | 58 (61.7) |  |
| Elevated | 152 (12.2) | 35 (11.3) | 31 (33.0) |  |
| Unknown | 125 (10.1) | 27 (8.7) | 5 (5.3) |  |
| **T stage** |  |  |  | 0.322 |
| T1 | 70 (5.6) | 15 (4.8) | 8 (8.5) |  |
| T2 | 116 (9.3) | 33 (10.6) | 3 (3.2) |  |
| T3 | 228 (18.4) | 51 (16.4) | 19 (20.2) |  |
| T4 | 828 (66.7) | 212 (68.2) | 64 (68.1) |  |
| **N stage** |  |  |  | <0.001 |
| N0 | 243 (19.6) | 54 (17.4) | 19 (20.2) |  |
| N1 | 269 (21.7) | 72 (23.2) | 8 (8.5) |  |
| N2 | 308 (24.8) | 69 (22.2) | 21 (22.3) |  |
| N3a | 308 (24.8) | 88 (28.3) | 44 (46.8) |  |
| N3b | 114 (9.2) | 28 (9.0) | 2 (2.1) |  |
| **Differentiation** |  |  |  | <0.001 |
| Low | 1112 (89.5) | 268 (86.2) | 66(70.2) |  |
| Middle/High | 130 (10.5) | 43 (13.8) | 28(29.8) |  |
| **Tumor location** |  |  |  | <0.001 |
| Fundus | 110 (8.9) | 35 (11.3) | 8 (8.5) |  |
| Body | 310 (25.0) | 85 (27.3) | 18 (19.1) |  |
| Antrum | 782 (63.0) | 184 (59.2) | 57 (60.6) |  |
| ≥2 locations | 40 (3.2) | 7 (2.3) | 11 (11.7) |  |
| **Lymph node ratio** | 0.3 ± 0.3 | 0.3 ± 0.3 | 0.3 ± 0.3 | 0.087 |
| **Gastrectomy** |  |  |  | 0.500 |
| Proximal | 10 (0.8) | 2 (0.6) | 1 (1.1) |  |
| Distal | 879 (70.8) | 210 (67.5) | 71 (75.5) |  |
| Total | 313 (25.2) | 85 (27.3) | 17 (18.1) |  |
| With organ resection | 40 (3.2) | 14 (4.5) | 5 (5.3) |  |
| **Adjuvant chemotherapy** |  |  |  | 0.100 |
| No | 298 (24.0) | 93 (29.9) | 24 (25.5) |  |
| Yes | 944 (76.0) | 218 (70.1) | 70 (74.5) |  |

**Notes:** Continuous variables are expressed as mean ± standard deviation and were compared using the one-way analysis of variance or the Kruskal-Wallis test. Categorical variables are expressed as count (percentage) and were compared using the Chi-square test. *P*-values < 0.05 were considered statistically significant. BMI, body mass index; CEA, carcinoembryonic antigen.

**Supplementary Table 4** Unadjusted and multivariable Cox regression analyses of factors predicting survival in patients with gastric cancer in the training cohort

| **Characteristic** | **RFS (unadjusted)** | |  | **RFS (multivariable)** | |  | **DSS (unadjusted)** | |  | **DSS (multivariable)** | |  |
| --- | --- | --- | --- | --- | --- | --- | --- | --- | --- | --- | --- | --- |
|  | HR (95% CI) | *P*-value |  | HR (95% CI) | *P*-value |  | HR (95% CI) | *P*-value |  | HR (95% CI) | *P*-value |  |
| **Model score** | **979.2**  **(566.8, 1691.9)** | **<0.001** |  | **607.4**  **(343.4, 1074.3)** | **<0.001** |  | **281.6**  **(162.7, 487.5)** | **<0.001** |  | **868.1**  **(461.4, 1633.1)** | **<0.001** |  |
| Age (years) |  |  |  |  |  |  |  |  |  |  |  |  |
| <65 | 1.0 (Ref.) |  |  |  |  |  | 1.0 (Ref.) |  |  | 1.0 (Ref.) |  |  |
| ≥65 | 1.1 (0.9, 1.3) | 0.631 |  |  |  |  | 1.3 (1.1, 1.6) | 0.015 |  | 1.2 (0.9, 1.5) | 0.194 |  |
| Sex |  |  |  |  |  |  |  |  |  |  |  |  |
| Male | 1.0 (Ref.) |  |  |  |  |  | 1.0 (Ref.) |  |  |  |  |  |
| Female | 1.2 (1.0, 1.4) | 0.127 |  |  |  |  | 1.0 (0.8, 1.3) | 0.739 |  |  |  |  |
| BMI (kg/m^2^) |  |  |  |  |  |  |  |  |  |  |  |  |
| <18.5 | 1.0 (Ref.) |  |  | 1.0 (Ref.) |  |  | 1.0 (Ref.) |  |  | 1.0 (Ref.) |  |  |
| 18.5–25 | 0.7 (0.6, 0.9) | 0.009 |  | 0.8 (0.6, 1.0) | 0.063 |  | 0.8 (0.6, 1.0) | 0.059 |  | 0.7 (0.5, 0.9) | 0.003 |  |
| ≥25 | 0.6 (0.4, 0.8) | 0.001 |  | 0.8 (0.5, 1.1) | 0.107 |  | 0.6 (0.4, 0.9) | 0.006 |  | 0.7 (0.5, 1.0) | 0.046 |  |
| CEA level |  |  |  |  |  |  |  |  |  |  |  |  |
| Normal | 1.0 (Ref.) |  |  | 1.0 (Ref.) |  |  | 1.0 (Ref.) |  |  | 1.0 (Ref.) |  |  |
| Elevated | 1.4 (1.1, 1.8) | 0.009 |  | 1.4 (1.1, 1.9) | 0.009 |  | 1.6 (1.3, 2.1) | <0.001 |  | 1.5 (1.2, 2.0) | 0.002 |  |
| Unknown | 1.0 (0.8, 1.4) | 0.887 |  | 0.8 (0.6, 1.1) | 0.201 |  | 1.1 (0.8, 1.5) | 0.658 |  | 1.0 (0.7, 1.4) | 0.995 |  |
| T stage |  |  |  |  |  |  |  |  |  |  |  |  |
| T1 | 1.0 (Ref.) |  |  | 1.0 (Ref.) |  |  | 1.0 (Ref.) |  |  | 1.0 (Ref.) |  |  |
| T2 | 1.1 (0.5, 2.1) | 0.852 |  | 0.9 (0.5, 1.9) | 0.867 |  | 1.4 (0.6, 3.1) | 0.378 |  | 1.1 (0.5, 2.4) | 0.858 |  |
| T3 | 2.0 (1.1, 3.7) | 0.020 |  | 1.8 (1.0, 3.3) | 0.058 |  | 2.2 (1.1, 4.4) | 0.034 |  | 1.5 (0.7, 3.1) | 0.272 |  |
| T4 | 2.9 (1.7, 5.0) | <0.001 |  | 1.8 (1.0, 3.2) | 0.038 |  | 3.8 (1.9, 7.3) | <0.001 |  | 1.7 (0.9, 3.4) | 0.110 |  |
| N stage |  |  |  |  |  |  |  |  |  |  |  |  |
| N0 | 1.0 (Ref.) |  |  | 1.0 (Ref.) |  |  | 1.0 (Ref.) |  |  | 1.0 (Ref.) |  |  |
| N1 | 1.1 (0.8, 1.5) | 0.691 |  | 1.3 (0.9, 1.8) | 0.212 |  | 1.0 (0.7, 1.5) | 0.893 |  | 1.0 (0.7, 1.5) | 0.991 |  |
| N2 | 1.5 (1.1, 2.0) | 0.011 |  | 1.5 (1.0, 2.1) | 0.033 |  | 1.4 (1.0, 1.9) | 0.048 |  | 1.1 (0.7, 1.6) | 0.748 |  |
| N3a | 3.1 (2.3, 4.1) | <0.001 |  | 1.8 (1.1, 2.8) | 0.012 |  | 2.9 (2.1, 3.9) | <0.001 |  | 1.1 (0.7, 1.8) | 0.595 |  |
| N3b | 4.3 (3.0, 6.1) | <0.001 |  | 1.6 (0.9, 2.9) | 0.142 |  | 4.6 (3.2, 6.6) | <0.001 |  | 1.1 (0.6, 2.0) | 0.839 |  |
| Differentiation |  |  |  |  |  |  |  |  |  |  |  |  |
| Low | 1.0 (Ref.) |  |  | 1.0 (Ref.) |  |  | 1.0 (Ref.) |  |  | 1.0 (Ref.) |  |  |
| Middle/High | 0.6 (0.5, 0.9) | 0.009 |  | 0.9 (0.6, 1.3) | 0.629 |  | 0.5 (0.3, 0.7) | 0.001 |  | 0.7 (0.4, 1.0) | 0.046 |  |
| Tumor location |  |  |  |  |  |  |  |  |  |  |  |  |
| Fundus | 1.0 (Ref.) |  |  | 1.0 (Ref.) |  |  | 1.0 (Ref.) |  |  | 1.0 (Ref.) |  |  |
| Body | 0.8 (0.6, 1.1) | 0.229 |  | 0.8 (0.6, 1.1) | 0.111 |  | 1.1 (0.8, 1.5) | 0.741 |  | 0.9 (0.7, 1.4) | 0.763 |  |
| Antrum | 0.5 (0.4, 0.6) | <0.001 |  | 0.5 (0.4, 0.7) | <0.001 |  | 0.6 (0.4, 0.9) | 0.005 |  | 0.7 (0.5, 0.9) | 0.019 |  |
| ≥2 locations | 1.8 (1.1, 2.9) | 0.014 |  | 1.1 (0.7, 1.9) | 0.595 |  | 2.4 (1.4, 4.0) | 0.001 |  | 0.8 (0.5, 1.4) | 0.426 |  |
| Lymph node ratio | 7.8 (5.7, 10.8) | <0.001 |  | 2.0 (1.0, 3.8) | 0.036 |  | 7.7 (5.5, 10.9) | <0.001 |  | 4.2 (2.1, 8.2) | <0.001 |  |
| Gastrectomy |  |  |  |  |  |  |  |  |  |  |  |  |
| Proximal | 1.0 (Ref.) |  |  |  |  |  | 1.0 (Ref.) |  |  |  |  |  |
| Distal | 0.6 (0.2, 1.6) | 0.286 |  |  |  |  | 2.2 (0.3, 15.8) | 0.426 |  |  |  |  |
| Total | 1.3 (0.5, 3.6) | 0.560 |  |  |  |  | 5.0 (0.7, 35.6) | 0.109 |  |  |  |  |
| With organ resection | 2.1 (0.8, 6.1) | 0.154 |  |  |  |  | 6.2 (0.8, 45.7) | 0.076 |  |  |  |  |
| Adjuvant chemotherapy |  |  |  |  |  |  |  |  |  |  |  |  |
| No | 1.0 (Ref.) |  |  | 1.0 (Ref.) |  |  | 1.0 (Ref.) |  |  | 1.0 (Ref.) |  |  |
| Yes | 0.7 (0.6, 0.9) | 0.002 |  | 0.7 (0.6, 0.9) | 0.002 |  | 0.5 (0.4, 0.6) | <0.001 |  | 0.5 (0.4, 0.7) | <0.001 |  |

BMI, body mass index; CI, confidence interval; DSS, disease-specific survival; HR, hazard ratio.; Ref., reference; RFS, recurrence-free survival.

**Supplementary Table 5** Unadjusted and multivariable Cox regression analyses of factors predicting survival in patients with gastric cancer in the internal validation cohort

| **Characteristic** | **RFS (unadjusted)** | |  | **RFS (multivariable)** | |  | **DSS (unadjusted)** | |  | **DSS (multivariable)** | |
| --- | --- | --- | --- | --- | --- | --- | --- | --- | --- | --- | --- |
|  | HR (95% CI) | *P*-value |  | HR (95% CI) | *P*-value |  | HR (95% CI) | *P*-value |  | HR (95% CI) | *P*-value |
| **Model score** | **328.7**  **(100.3, 1077.7)** | **<0.001** |  | **173.2**  **(45.7, 656.7)** | **<0.001** |  | **148.0**  **(44.2, 495.1)** | **<0.001** |  | **794.7**  **(163.9, 3854.3)** | **<0.001** |
| Age (years) |  |  |  |  |  |  |  |  |  |  |  |
| <65 | 1.0 (Ref.) |  |  |  |  |  | 1.0 (Ref.) |  |  |  |  |
| ≥65 | 1.3 (0.8, 1.9) | 0.272 |  |  |  |  | 1.3 (0.8, 1.9) | 0.260 |  |  |  |
| Sex |  |  |  |  |  |  |  |  |  |  |  |
| Male | 1.0 (Ref.) |  |  |  |  |  | 1.0 (Ref.) |  |  |  |  |
| Female | 0.8 (0.6, 1.2) | 0.377 |  |  |  |  | 0.8 (0.5, 1.2) | 0.293 |  |  |  |
| BMI (kg/m^2^) |  |  |  |  |  |  |  |  |  |  |  |
| <18.5 | 1.0 (Ref.) |  |  |  |  |  | 1.0 (Ref.) |  |  |  |  |
| 18.5–25 | 0.8 (0.4, 1.3) | 0.306 |  |  |  |  | 0.7 (0.4, 1.2) | 0.229 |  |  |  |
| ≥25 | 0.7 (0.4, 1.4) | 0.352 |  |  |  |  | 0.7 (0.3, 1.4) | 0.282 |  |  |  |
| CEA level |  |  |  |  |  |  |  |  |  |  |  |
| Normal | 1.0 (Ref.) |  |  |  |  |  | 1.0 (Ref.) |  |  |  |  |
| Elevated | 1.3 (0.7, 2.2) | 0.379 |  |  |  |  | 1.3 (0.7, 2.2) | 0.393 |  |  |  |
| Unknown | 0.5 (0.2, 1.1) | 0.088 |  |  |  |  | 0.6 (0.3, 1.3) | 0.212 |  |  |  |
| T stage |  |  |  |  |  |  |  |  |  |  |  |
| T1 | 1.0 (Ref.) |  |  |  |  |  | 1.0 (Ref.) |  |  |  |  |
| T2 | 0.4 (0.1, 2.2) | 0.317 |  |  |  |  | 0.4 (0.1, 2.2) | 0.324 |  |  |  |
| T3 | 1.4 (0.4, 4.9) | 0.648 |  |  |  |  | 1.5 (0.4, 5.5) | 0.536 |  |  |  |
| T4 | 3.0 (1.0, 9.5) | 0.060 |  |  |  |  | 2.7 (0.9, 8.6) | 0.086 |  |  |  |
| N stage |  |  |  |  |  |  |  |  |  |  |  |
| N0 | 1.0 (Ref.) |  |  | 1.0 (Ref.) |  |  | 1.0 (Ref.) |  |  | 1.0 (Ref.) |  |
| N1 | 0.7 (0.3, 1.7) | 0.476 |  | 0.7 (0.3, 1.7) | 0.458 |  | 0.7 (0.3, 1.6) | 0.380 |  | 0.6 (0.3, 1.5) | 0.301 |
| N2 | 2.0 (1.0, 4.1) | 0.047 |  | 1.0 (0.4, 2.3) | 0.967 |  | 2.0 (1.0, 4.0) | 0.060 |  | 0.7 (0.3, 1.6) | 0.404 |
| N3a | 3.5 (1.8, 6.7) | <0.001 |  | 1.4 (0.5, 3.6) | 0.489 |  | 3.1 (1.6, 6.1) | 0.001 |  | 0.6 (0.2, 1.7) | 0.334 |
| N3b | 7.0 (3.4, 14.5) | <0.001 |  | 1.0 (0.3, 3.8) | 0.981 |  | 5.6 (2.7, 11.7) | <0.001 |  | 0.2 (0.0, 0.7) | 0.014 |
| Differentiation |  |  |  |  |  |  |  |  |  |  |  |
| Low | 1.0 (Ref.) |  |  |  |  |  | 1.0 (Ref.) |  |  |  |  |
| Middle/High | 0.6 (0.3, 1.2) | 0.163 |  |  |  |  | 0.7 (0.4, 1.3) | 0.248 |  |  |  |
| Tumor location |  |  |  |  |  |  |  |  |  |  |  |
| Fundus | 1.0 (Ref.) |  |  | 1.0 (Ref.) |  |  | 1.0 (Ref.) |  |  |  |  |
| Body | 1.1 (0.6, 2.0) | 0.807 |  | 0.7 (0.4, 1.4) | 0.308 |  | 1.2 (0.6, 2.2) | 0.636 |  |  |  |
| Antrum | 0.6 (0.4, 1.2) | 0.138 |  | 0.6 (0.3, 1.1) | 0.086 |  | 0.6 (0.4, 1.2) | 0.150 |  |  |  |
| ≥2 locations | 3.1 (1.1, 8.2) | 0.026 |  | 0.6 (0.2, 1.7) | 0.340 |  | 2.6 (0.8, 7.9) | 0.101 |  |  |  |
| Lymph node ratio | 17.4 (9.2, 33.2) | <0.001 |  | 6.0 (1.5, 23.6) | 0.010 |  | 12.5 (6.4, 24.4) | <0.001 |  | 35.4 (7.4, 169.4) | <0.001 |
| Gastrectomy |  |  |  |  |  |  |  |  |  |  |  |
| Proximal | 1.0 (Ref.) |  |  |  |  |  | 1.0 (Ref.) |  |  |  |  |
| Distal | 0.5 (0.1, 3.8) | 0.517 |  |  |  |  | 0.6 (0.1, 4.0) | 0.563 |  |  |  |
| Total | 1.0 (0.1, 7.0) | 0.969 |  |  |  |  | 1.0 (0.1, 7.3) | 0.995 |  |  |  |
| With organ resection | 1.7 (0.2, 13.3) | 0.611 |  |  |  |  | 1.9 (0.2, 14.6) | 0.555 |  |  |  |
| Adjuvant chemotherapy |  |  |  |  |  |  |  |  |  |  |  |
| No | 1.0 (Ref.) |  |  |  |  |  | 1.0 (Ref.) |  |  |  |  |
| Yes | 0.8 (0.5, 1.1) | 0.202 |  |  |  |  | 0.7 (0.5, 1.0) | 0.063 |  |  |  |

BMI, body mass index; CI, confidence interval; DSS, disease-specific survival; HR, hazard ratio.; Ref., reference; RFS, recurrence-free survival.

**Supplementary Table 6** Unadjusted and multivariable Cox regression analyses of factors predicting survival in patients with gastric cancer in the external validation cohort

| **Characteristic** | **RFS (unadjusted)** | |  | **RFS (multivariable)** | | |  | **DSS (unadjusted)** | | |  | **DSS (multivariable)** | |
| --- | --- | --- | --- | --- | --- | --- | --- | --- | --- | --- | --- | --- | --- |
|  | HR (95% CI) | *P*-value |  | HR (95% CI) | *P*-value | |  | HR (95% CI) | *P*-value | |  | HR (95% CI) | *P*-value |
| **Model score** | **77.2**  **(10.3, 581.0)** | **<0.001** |  | **46.7**  **(5.6, 392.5)** | | **<0.001** |  | **71.0**  **(9.4, 537.3)** | | **<0.001** |  | **329.8**  **(27.8, 3910.0)** | **<0.001** |
| Age (years) |  |  |  |  | |  |  |  | |  |  |  |  |
| <65 | 1.0 (Ref.) |  |  |  | |  |  | 1.0 (Ref.) | |  |  |  |  |
| ≥65 | 0.9 (0.4, 2.0) | 0.804 |  |  | |  |  | 1.0 (0.4, 2.1) | | 0.907 |  |  |  |
| Sex |  |  |  |  | |  |  |  | |  |  |  |  |
| Male | 1.0 (Ref.) |  |  |  | |  |  | 1.0 (Ref.) | |  |  |  |  |
| Female | 1.1 (0.6, 2.4) | 0.718 |  |  | |  |  | 1.2 (0.6, 2.4) | | 0.693 |  |  |  |
| BMI (kg/m^2^) |  |  |  |  | |  |  |  | |  |  |  |  |
| <18.5 | 1.0 (Ref.) |  |  |  | |  |  | 1.0 (Ref.) | |  |  |  |  |
| 18.5–25 | 2.3 (0.3, 17.2) | 0.412 |  |  | |  |  | 2.2 (0.3, 16.1) | | 0.450 |  |  |  |
| ≥25 | 6.8 (0.8, 54.2) | 0.072 |  |  | |  |  | 6.3 (0.8, 50.9) | | 0.082 |  |  |  |
| CEA level |  |  |  |  | |  |  |  | |  |  |  |  |
| Normal | 1.0 (Ref.) |  |  |  | |  |  | 1.0 (Ref.) | |  |  |  |  |
| Elevated | 1.2 (0.5, 2.6) | 0.671 |  |  | |  |  | 1.2 (0.5, 2.5) | | 0.711 |  |  |  |
| Unknown | 2.3 (0.8, 6.8) | 0.139 |  |  | |  |  | 2.3 (0.8, 6.9) | | 0.128 |  |  |  |
| T stage |  |  |  |  | |  |  |  | |  |  |  |  |
| T1 | 1.0 (Ref.) |  |  |  | |  |  | 1.0 (Ref.) | |  |  |  |  |
| T2 | 2.8 (0.4, 20.0) | 0.302 |  |  | |  |  | 2.7 (0.4, 19.3) | | 0.319 |  |  |  |
| T3 | 2.0 (0.4, 10.3) | 0.425 |  |  | |  |  | 2.0 (0.4, 10.7) | | 0.398 |  |  |  |
| T4 | 1.7 (0.4, 7.2) | 0.476 |  |  | |  |  | 1.6 (0.4, 7.0) | | 0.503 |  |  |  |
| N stage |  |  |  |  | |  |  |  | |  |  |  |  |
| N0 | 1.0 (Ref.) |  |  |  | |  |  | 1.0 (Ref.) | |  |  |  |  |
| N1 | 1.9 (0.3, 10.3) | 0.475 |  |  | |  |  | 1.9 (0.3, 10.7) | | 0.453 |  |  |  |
| N2 | 1.3 (0.4, 4.5) | 0.701 |  |  | |  |  | 1.3 (0.4, 4.5) | | 0.716 |  |  |  |
| N3a | 2.5 (0.9, 7.5) | 0.094 |  |  | |  |  | 2.5 (0.9, 7.5) | | 0.095 |  |  |  |
| N3b | NA | NA |  |  | |  |  | NA | | NA |  |  |  |
| Differentiation |  |  |  |  | |  |  |  | |  |  |  |  |
| Low | 1.0 (Ref.) |  |  |  | |  |  | 1.0 (Ref.) | |  |  |  |  |
| Middle/High | 0.9 (0.4, 2.0) | 0.745 |  |  | |  |  | 0.9 (0.4, 2.0) | | 0.788 |  |  |  |
| Tumor location |  |  |  |  | |  |  |  | |  |  |  |  |
| Fundus | 1.0 (Ref.) |  |  | 1.0 (Ref.) | |  |  | 1.0 (Ref.) | |  |  | 1.0 (Ref.) |  |
| Body | 2.5 (0.5, 11.7) | 0.258 |  | 2.4 (0.5, 12) | | 0.288 |  | 2.5 (0.5, 11.8) | | 0.257 |  | 1.2 (0.2, 6.1) | 0.867 |
| Antrum | 1.3 (0.3, 5.9) | 0.721 |  | 1.9 (0.4, 8.4) | | 0.415 |  | 1.4 (0.3, 6.1) | | 0.689 |  | 0.6 (0.1, 2.9) | 0.559 |
| ≥2 locations | 7.1 (1.4, 35.0) | 0.016 |  | 7.5 (1.5, 37.1) | | 0.013 |  | 6.8 (1.4, 33.2) | | 0.018 |  | 4.1 (0.8, 20.8) | 0.092 |
| Lymph node ratio | 6.9 (2.0, 23.3) | 0.002 |  | 5.7 (1.5, 22.1) | | 0.013 |  | 7.0 (2.1, 23.5) | | 0.002 |  | 4.8 (1.2, 19.3) | 0.028 |
| Gastrectomy |  |  |  |  | |  |  |  | |  |  |  |  |
| Proximal | 1.0 (Ref.) |  |  |  | |  |  | 1.0 (Ref.) | |  |  |  |  |
| Distal | NA | NA |  |  | |  |  | NA | | NA |  |  |  |
| Total | NA | NA |  |  | |  |  | NA | | NA |  |  |  |
| With organ resection | NA | NA |  |  | |  |  | NA | | NA |  |  |  |
| Adjuvant chemotherapy |  |  |  |  | |  |  |  | |  |  |  |  |
| No | 1.0 (Ref.) |  |  |  | |  |  | 1.0 (Ref.) | |  |  |  |  |
| Yes | 0.9 (0.4, 2.1) | 0.782 |  |  | |  |  | 0.9 (0.4, 2.0) | | 0.730 |  |  |  |

BMI, body mass index; CI, confidence interval; DSS, disease-specific survival; HR, hazard ratio.; Ref., reference; RFS, recurrence-free survival.

**Supplementary Table 7** Performance of different models for predicting recurrence-free survival

| **Models** | **AUC (95%CI)** | | | | | |
| --- | --- | --- | --- | --- | --- | --- |
|  | **Training cohort** | ***P*-value** | **Internal validation cohort** | ***P*-value** | **External validation cohort** | ***P*-value** |
| **Clinical** | 0.798 (0.776-0.815) | - | 0.735 (0.722-0.743) | - | 0.602 (0.586-0.617) | - |
| **SMDL** | 0.939 (0.922-0.953) | <0.001 | 0.858 (0.843-0.871) | <0.001 | 0.791 (0.776-0.802) | <0.001 |
| **Integrated** | 0.984 (0.963-0.993) | <0.001 | 0.876 (0.860-0.887) | <0.001 | 0.823 (0.809-0.836) | <0.001 |
| **Integrated vs Clinical** |  |  |  |  |  |  |
| **NRI** | 0.418 (0.301-0.565) | <0.001 | 0.273 (0.156-0.382) | <0.001 | 0.209 (0.120-0.341) | <0.001 |
| **IDI** | 0.201 (0.113-0.351) | <0.001 | 0.164 (0.087-0.296) | <0.001 | 0.148 (0.091-0.285) | <0.001 |

AUC, area under the curve; IDI, integrated discrimination improvement; NRI, net reclassification improvement.

**Supplementary Table 8** Performance of different models for predicting disease-specific survival

| **Model** | **AUC (95%CI)** | | | | | |
| --- | --- | --- | --- | --- | --- | --- |
|  | **Training cohort** | ***P*-value** | **Internal validation cohort** | ***P*-value** | **External validation cohort** | ***P*-value** |
| **Clinical** | 0.792 (0.768-0.809) | - | 0.722 (0.708-0.736) | - | 0.606 (0.592-0.619) | - |
| **SMDL** | 0.943 (0.926-0.957) | <0.001 | 0.841 (0.825-0.853) | <0.001 | 0.806 (0.791-0.818) | <0.001 |
| **Integrated** | 0.966 (0.952-0.976) | <0.001 | 0.859 (0.837-0.875) | <0.001 | 0.822 (0.807-0.839) | <0.001 |
| **Integrated vs Clinical** |  |  |  |  |  |  |
| NRI | 0.422 (0.253-0.606) | <0.001 | 0.213 (0.101-0.386) | <0.001 | 0.259 (0.129-0.427) | <0.001 |
| IDI | 0.176 (0.108-0.293) | <0.001 | 0.108 (0.061-0.198) | <0.001 | 0.127 (0.068-0.279) | <0.001 |

AUC, area under the curve; IDI, integrated discrimination improvement; NRI, net reclassification improvement.

**Supplementary Figures**


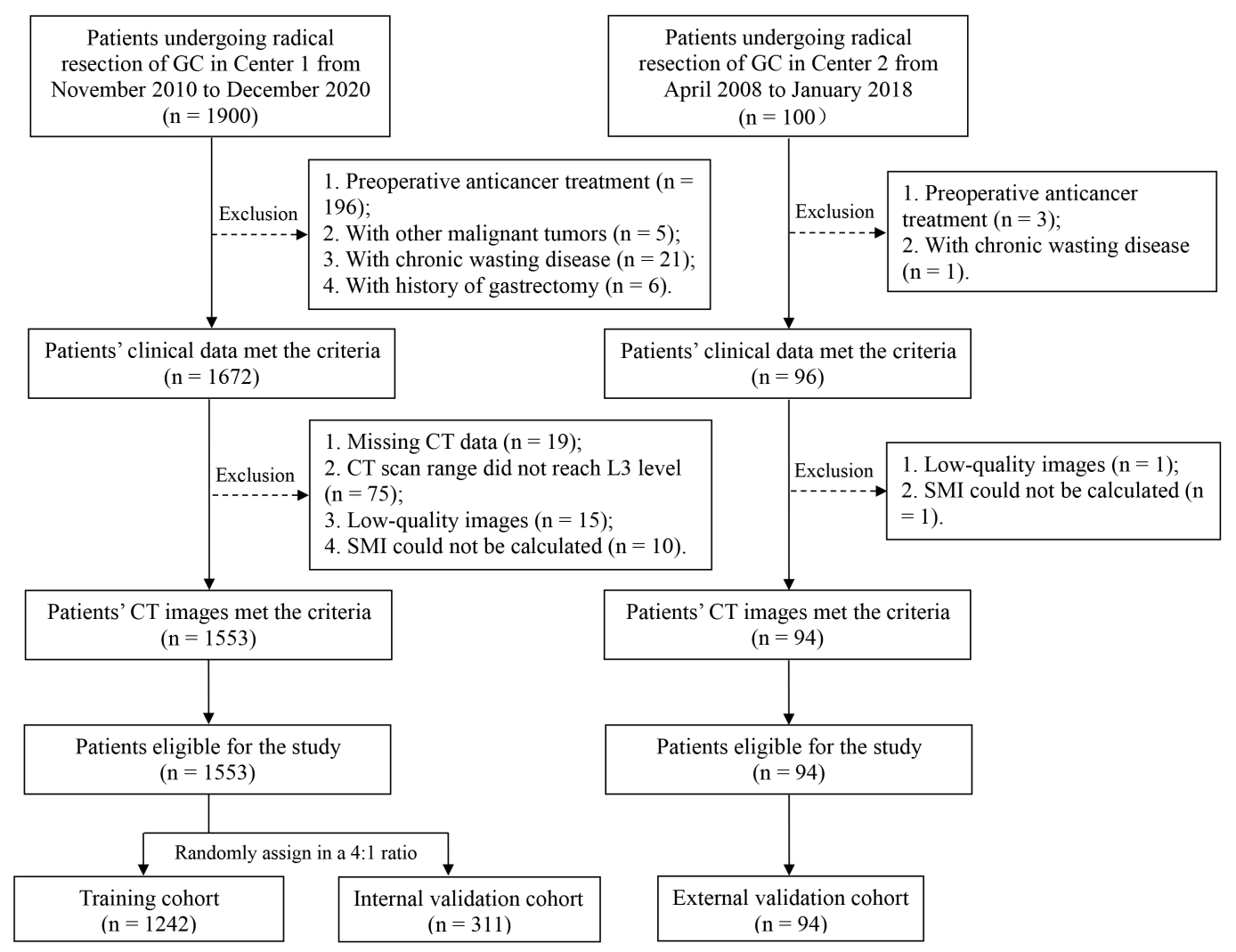


**Supplementary Fig. 1** Study flowchart showing inclusions and exclusions. GC, gastric cancer; SMI, skeletal muscle index.


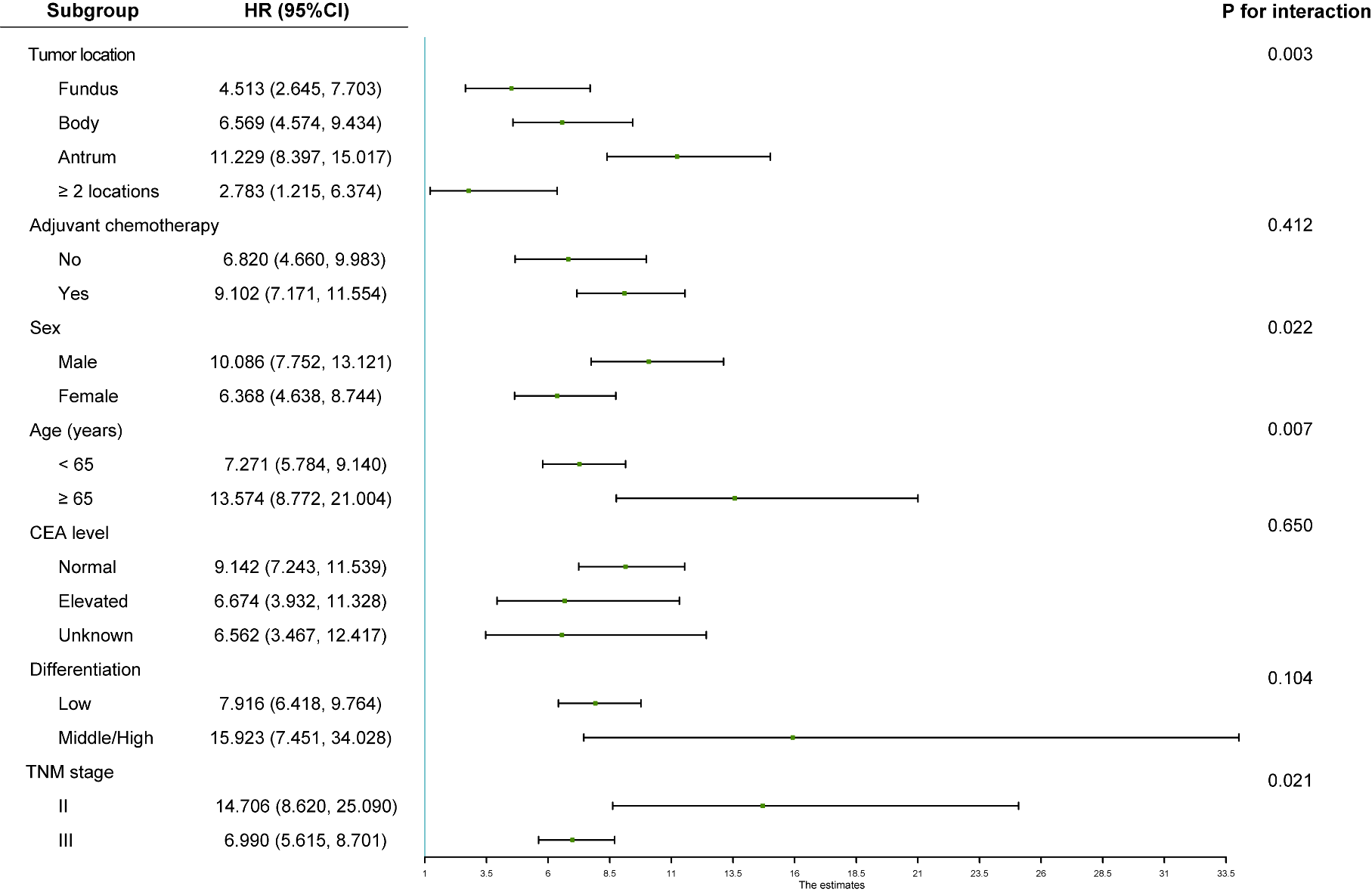


**Supplementary Fig. 2** Forest plots of subgroup analysis of the association between model score and recurrence-free survival


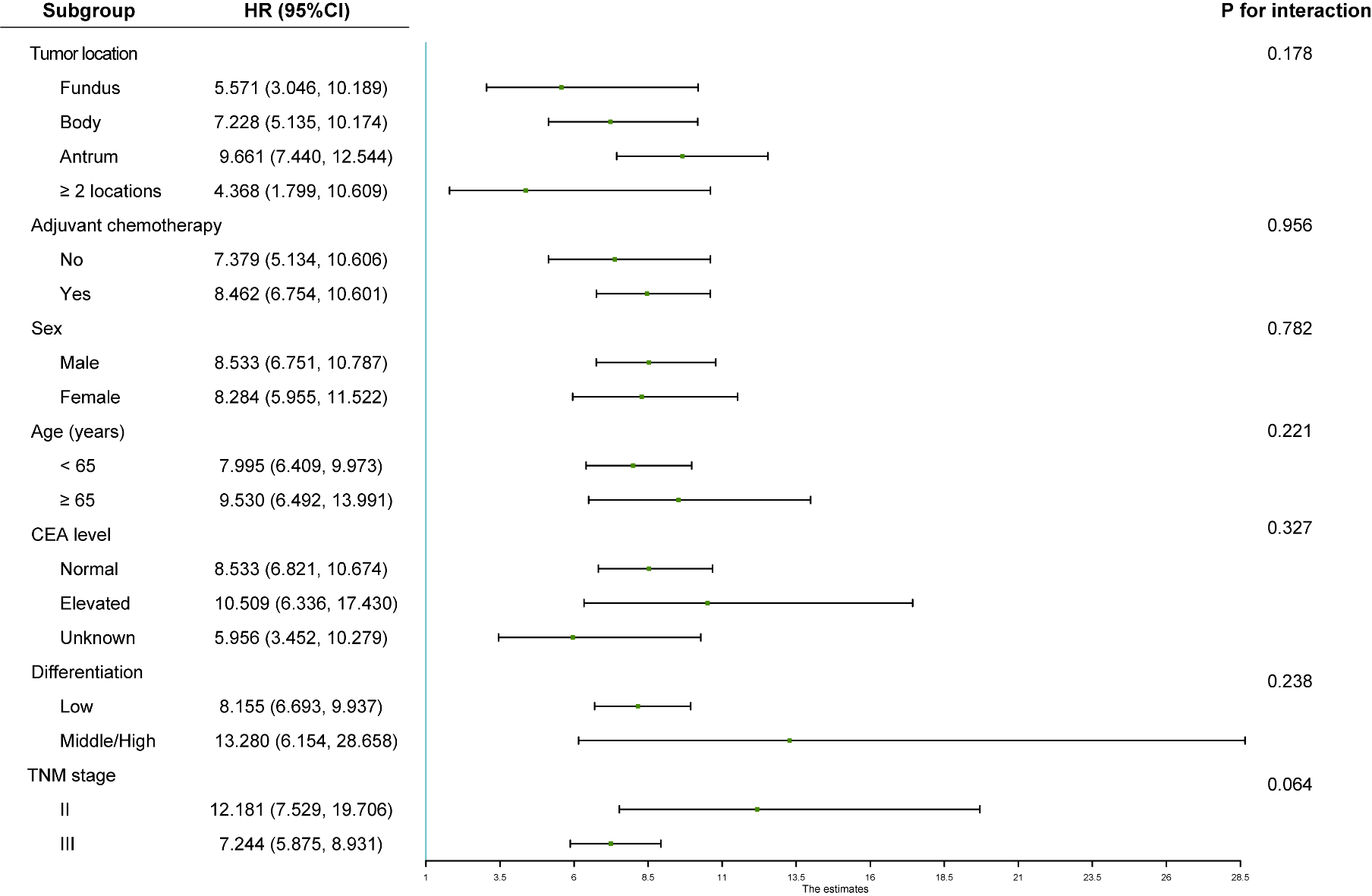


**Supplementary Fig. 3** Forest plots of subgroup analysis of the association between model score and disease-specific survival
